# Supplementary material for: Continuous Fixed-Bed Column Studies on Congo Red Dye Adsorption-Desorption Using Free and Immobilized Nelumbo nucifera Leaf Adsorbent
Source: Polymers (Basel). 2021 Dec 24;14(1):54. doi: 10.3390/polym14010054 (PMC8747315; doi:10.3390/polym14010054)
Supplement: Supplementary file 1 [file polymers-14-00054-s001.zip › polymers-1485236-supplementary.pdf]

polymers

Article

Continuous Fixed-Bed Column Studies on Congo Red Dye Adsorption-Desorption Using Free and Immobilized *Nelumbo nucifera* Leaf *Adsorbent*

Vairavel Parimelazhagan [1]*, Gautham Jeppu [1] and Nakul Rampal [2]

Supplementary Materials

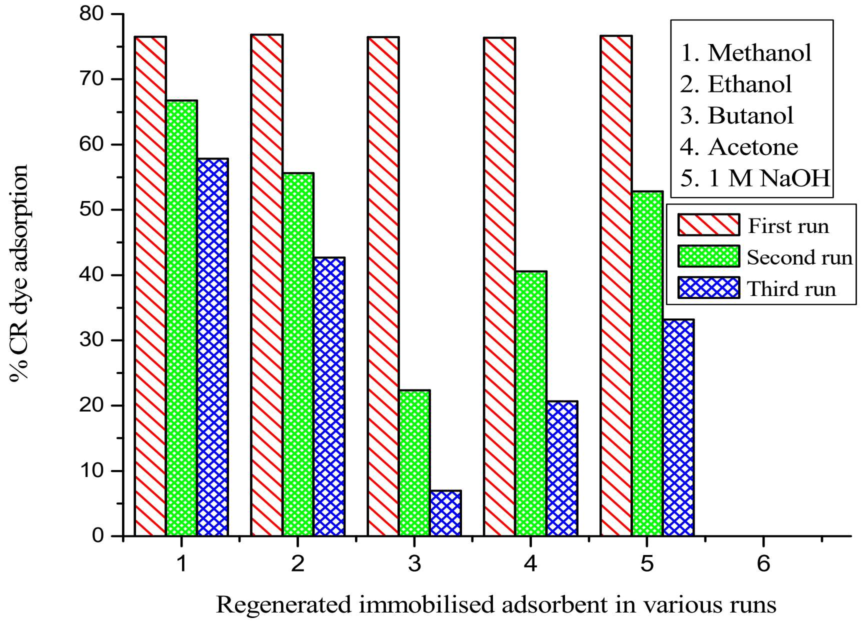

Figure S1. Reusability of immobilized NNLP adsorbent for the adsorption of CR dye in various runs. (Initial pH: 6; initial dye concentration: 200 mg L^{-1}; volume of dye solution: 100 mL; agitation speed: 150 rpm; temperature: 301 K; contact time: 24 h).

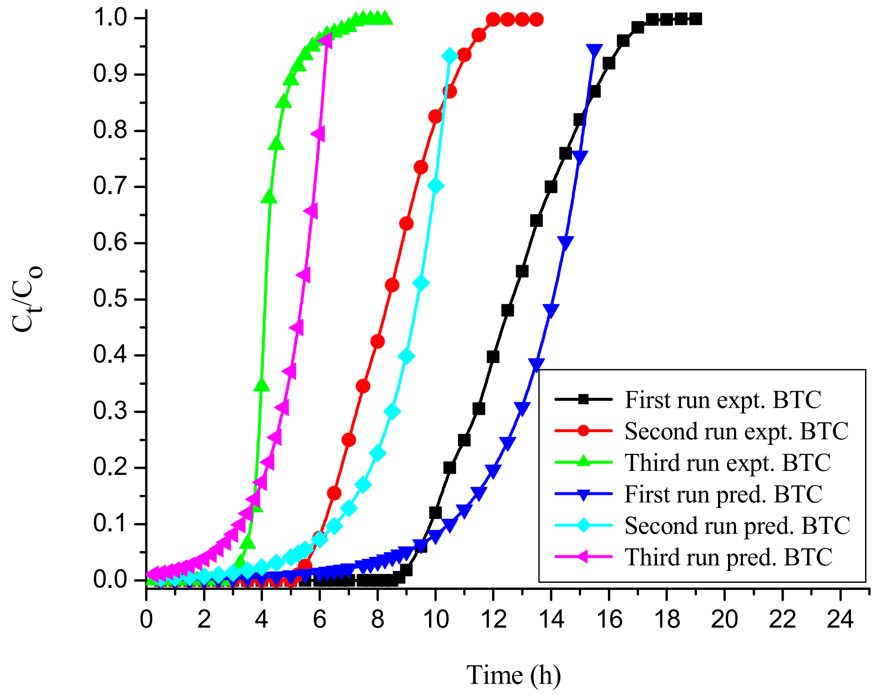

Figure S2. BTCs for experimental vs. simulated Adams-Bohart model for the decolorization of CR by free NNLP adsorbent in various runs (Initial pH: 6; flow rate: 1 mL min^{-1}; inlet dye concentration: 15 mg L^{-1}; temperature: 301 K).

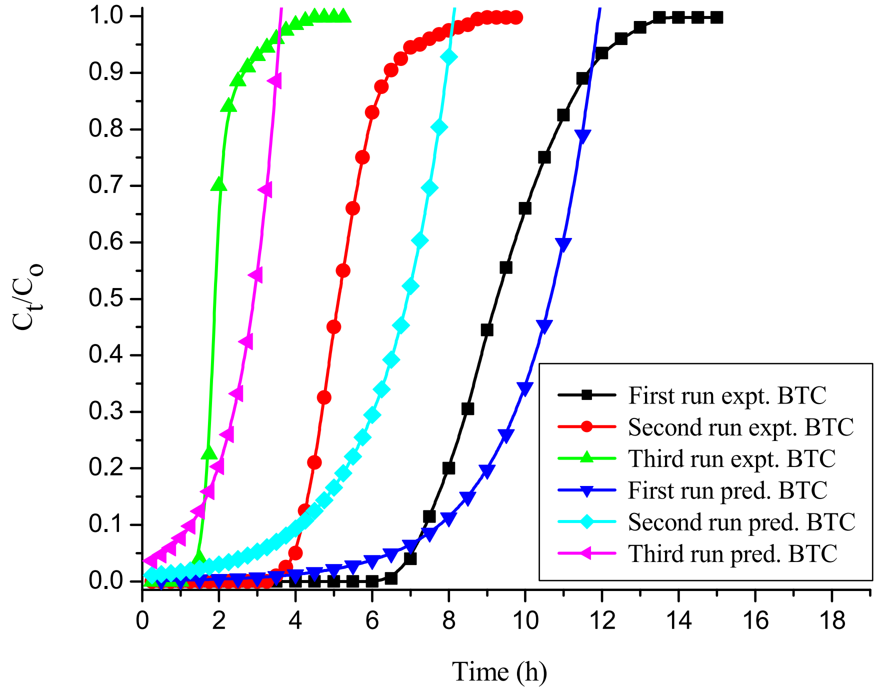

Figure S3. BTCs for experimental vs. simulated Adams-Bohart model for the decolorization of CR dye by sodium silicate gel immobilized NNLLP adsorbent in various runs (Initial pH: 6; flow rate: 1 mL min^{-1}; inlet dye concentration: 15 mg L^{-1}; temperature: 301 K).

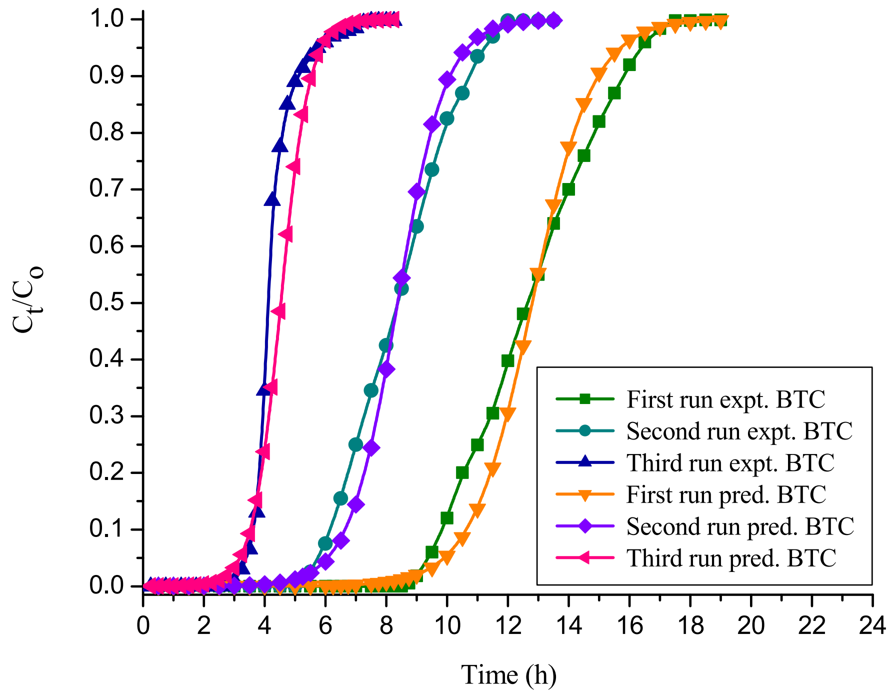

Figure S4. BTCs for experimental vs. simulated BDST model for the decolorization of CR by free NNLP adsorbent in various runs (Initial pH: 6; flow rate: 1 mL min^{-1}; inlet dye concentration: 15 mg L^{-1}; temperature: 301 K).

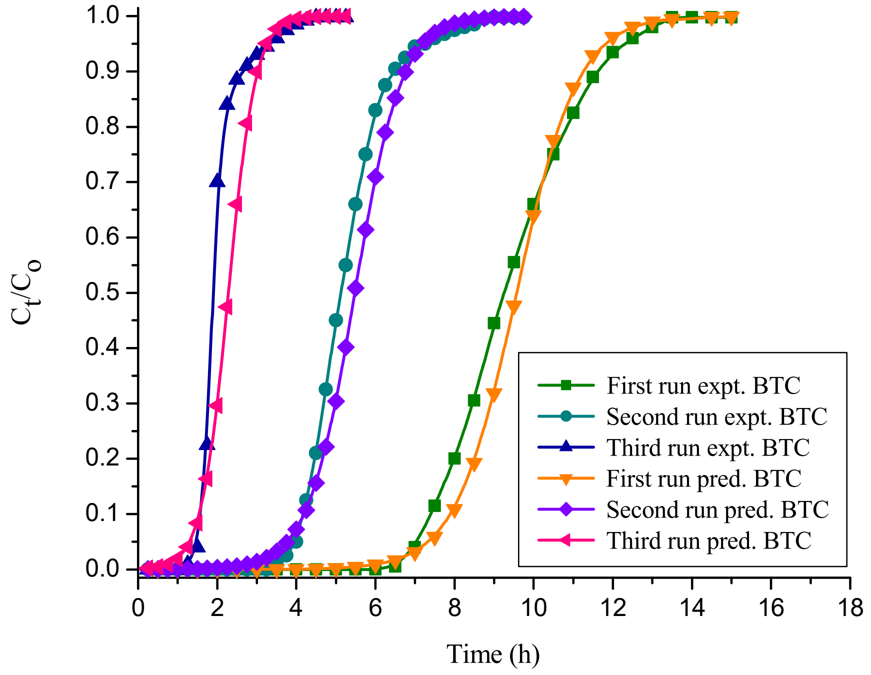

Figure S5. BTCs for experimental vs. simulated BDST model for the decolorization of CR dye by sodium silicate gel immobilized NNLP adsorbent in various runs (Initial pH: 6; flow rate: 1 mL min^{-1}; inlet dye concentration: 15 mg L^{-1}; temperature: 301 K).

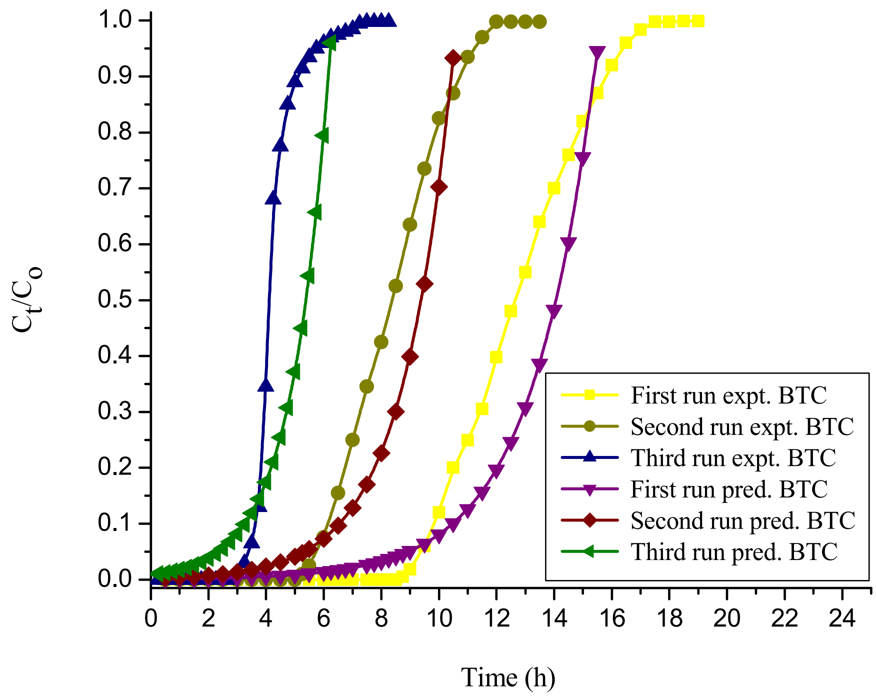

Figure S6. BTCs for experimental vs. simulated Wolborska model for the decolorization of CR by free NNLP adsorbent in various runs (Initial pH: 6; flow rate: 1 mL min^{-1}; inlet dye concentration: 15 mg L^{-1}; temperature: 301 K).

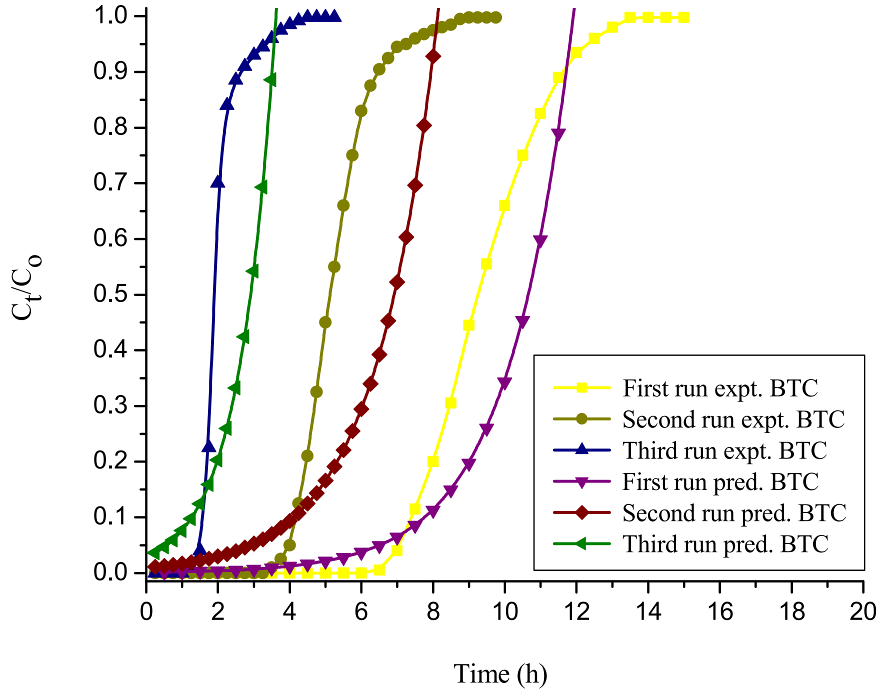

Figure S7. BTCs for experimental vs. simulated Wolborska model for the decolorization of CR dye by sodium silicate gel immobilized NNLP adsorbent in various runs (Initial pH:6; flow rate: 1 mL min^{-1}; inlet dye concentration: 15 mg L^{-1}; temperature: 301 K).

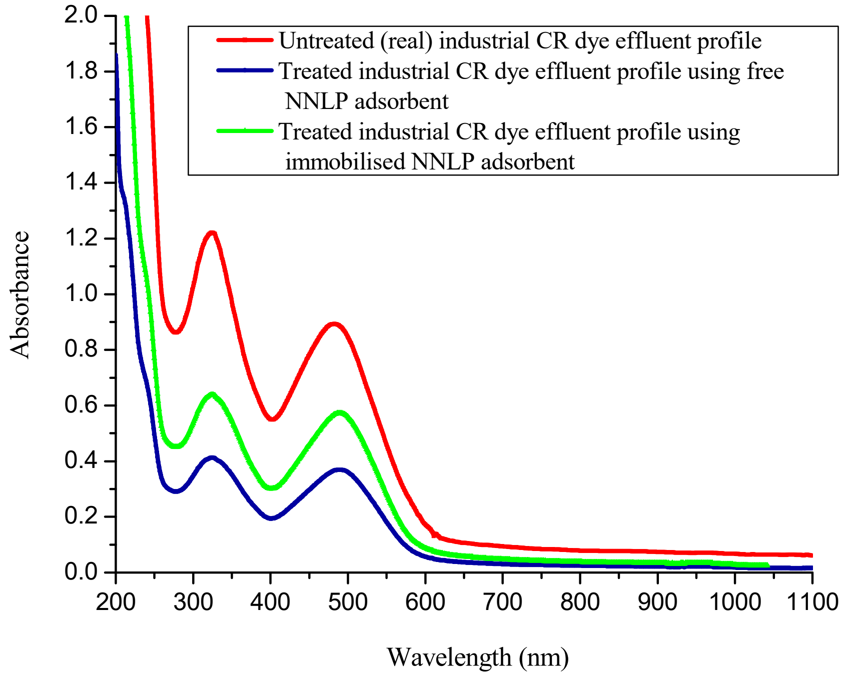

Figure S8. Industrial CR dye effluent decolorization profile obtained in column studies using free and immobilized NNLP adsorbents with untreated effluent profile (Initial pH: 6; bed height: 2.5 cm; flow rate: 1 mL min^{-1}; inlet adsorbate concentration: 215 mg L^{-1}; temperature: 301 K).

Table S1. Effect of adsorbent composition in various polymeric matrices for CR dye adsorption (Initial pH;6; initial dye concentration: 250 mg L^{-1}; free NNLP adsorbent particle size: 94 μm; immobilized NNLP adsorbent dosage: 6 g L^{-1}; agitation speed: 150 rpm; temperature: 301 K; contact time: 24 h).

NNLP adsorbent loading % (w/v)	% color removal of CR dye in various immobilized polymeric matrices			
	Calcium alginate	Polyvinyl alcohol	Polysulfone	Sodium silicate
1	70.39	62.24	69.84	75.48
2	71.65	65.38	72.35	80.26
3	73.47	68.54	74.16	83.64
4	75.58	69.48	75.90	81.28
5	76.83	67.82	77.24	78.34
6	74.92	65.16	78.56	74.12
7	72.67	62.98	75.62	69.76
8	70.29	60.35	71.18	65.52
9	68.04	58.08	66.45	60.34
10	65.24	56.34	62.58	54.26

Table S2. Desorption studies for the removal of CR dye from sodium silicate gel immobilized NNLP adsorbent in various runs.

Sl. No	Desorbing reagent	% desorption of CR dye from NNLP adsorbent		
		1st run	2nd run	3rd run
1	Methanol	72.870	61.243	52.670
2	Ethanol	63.961	47.655	34.822
3	Butanol	35.479	15.836	1.548
4	Acetone	49.660	33.258	11.367
5	1 M NaOH	58.148	43.912	25.784

Table S3. Reusability of sodium silicate gel immobilized NNLP adsorbent for the decolorization of CR dye in various runs.

Sl. No	Desorbing reagent	% color removal by regenerated immobilized NNLP adsorbent		
		1st run	2nd run	3rd run
1	Methanol	76.542	66.753	57.840
2	Ethanol	76.822	55.637	42.765
3	Butanol	76.485	22.342	6.978
4	Acetone	76.394	40.564	20.652
5	1 M NaOH	76.652	52.845	33.184
